# Effects of Microalgae as Biostimulants on Plant Growth, Content of Antioxidant Molecules and Total Antioxidant Capacity in *Chenopodium quinoa* Exposed to Salt Stress

**DOI:** 10.3390/plants14050781

**Published:** 2025-03-04

**Authors:** Sofia Fiorentino, Lorenza Bellani, Marco Santin, Antonella Castagna, Maria Cristina Echeverria, Lucia Giorgetti

**Affiliations:** 1 Institute of Biology and Agricultural Biotechnology (IBBA), National Research Council, Pisa Unit, 56124 Pisa, Italy; sofia.fiorentino@ibba.cnr.it (S.F.); lorenza.bellani@unisi.it (L.B.); 2 Department of Life Sciences, University of Siena, 53100 Siena, Italy; 3 Department of Agriculture, Food and Environment (DAFE), University of Pisa, 56124 Pisa, Italy; marco.santin@unipi.it (M.S.); antonella.castagna@unipi.it (A.C.); 4 eCIER Research Group, Department of Biotechnology, Universidad Técnica del Norte, Av. 17 de Julio 5–21 y Gral. José María Córdova, Ibarra 100150, Ecuador; mecheverria@utn.edu.ec

**Keywords:** antioxidants and antioxidant activity, biostimulants, salt stress, sprouts, quinoa (*Chenopodium quinoa* Willd.)

## Abstract

*Chenopodium quinoa* Willd. is a halophytic plant valued for its nutritional and nutraceutical properties, as well as its adaptability to diverse soil and climatic conditions. Biostimulant application enhances plant quality and resilience under adverse environmental conditions. The effects of microalgae extracts (*Ettlia pseudoalveolaris* and *Chlorella vulgaris*) and salt stress (NaCl 100, 200, 300 mM) were evaluated on 7-day-old seedlings of two quinoa varieties, ‘Tunkahuan’ and ‘Regalona’. The analysis focused on the content of antioxidant molecules (total phenolics and flavonoids), total antioxidant capacity (measured by DPPH, 2,2-Diphenyl-1-picrylhydrazyl, and FRAP, Ferric Reducing Antioxidant Power, assays), reactive oxygen species (ROS), the levels of lutein, β-carotene, chlorophyll *a* and *b*. Microalgae extracts and salt stress treatments significantly increased antioxidant molecules in both quinoa varieties. The highest antioxidant activity, measured by the DPPH assay, was observed in ‘Regalona’, while a dose-dependent increase in antioxidant capacity, by the FRAP assay, was evident in ‘Tunkahuan’ treated with Ettlia. ROS level was reduced by Ettlia in ‘Tunkahuan’ but not in ‘Regalona’. Pigment content increased with higher salt concentrations but decreased with the addition of biostimulants. These findings suggest that the application of microalgae extracts enhances bioactive compounds, improving salinity resistance and increasing the nutraceutical value of quinoa sprouts.

## 1. Introduction

One of the most pressing challenges of our time is addressing climate change, which requires a dual approach: adapting to new, sustainable lifestyles that prioritize reducing energy consumption and mitigating the damage inflicted on agricultural systems. Climate change, characterized by rising temperatures and an increase in extreme weather events, has far-reaching consequences for the entire planet. It profoundly impacts natural ecosystems and agricultural productivity, affecting both plant and animal life. Global warming and increased water evaporation in agricultural soils contribute to rising soil salinity, which adversely affects the early stages of plant development and ultimately reduces crop yields [1]. Elevated salinity leads to the accumulation of Na⁺ and Cl⁻ ions in plant tissues, resulting in morphological impairments and nutritional imbalances. This is primarily due to the competition between Na⁺ and K⁺ ions, which disrupts potassium assimilation. Additionally, salt stress triggers the accumulation of reactive oxygen species (ROS), which degrade chlorophyll and damage proteins and lipids in thylakoid membranes and photosystems. This, in turn, reduces chloroplast activity and overall photosynthetic efficiency [2,3].

Numerous studies have demonstrated that microalgal biostimulants can enhance plant resilience to salt stress by regulating various physiological processes, including antioxidant responses and photosynthetic efficiency [4]. Specifically, microalgae extracts protect chlorophyll from degradation by improving the uptake of essential cations such as Mg^2^⁺ and Fe^2^⁺, which are critical for chlorophyll synthesis. Furthermore, these extracts promote the accumulation of compatible solutes like proline, which functions as both an antioxidant and an osmoprotectant. Proline helps prevent damage to cell membranes and proteins, thereby strengthening cellular defenses against salt stress [5].

The application of algae extracts can mitigate the detrimental effects of salt stress by reducing the absorption of Na⁺ and promoting the uptake of K⁺. This helps maintain cellular osmotic potential and prevents Na⁺ from reaching toxic concentrations within the plant [6]. Additionally, algae extracts positively influence the biological activities of plant cells, enhancing seed germination and improving plant growth parameters. These benefits are attributed to the rich content of micro- and macro-elements, vitamins, antioxidant compounds, auxins, proline, mannitol, and amino acids present in the extracts [7].

Microalgal biostimulants are particularly advantageous due to the rapid growth and high biomass production of microalgae under a wide range of environmental conditions, including variations in temperature, light, humidity, and seasonal changes [7]. Unicellular microalgae, such as *Chlorella vulgaris*, are well-known for their biostimulant properties, especially in enhancing plant resilience under stress conditions [8]. However, there is a vast diversity of microalgae species, many of which remain uncharacterized, that hold potential for biostimulant applications. These species offer unique opportunities for research due to their adaptability to extreme environments and their untapped biodiversity [4]. Among these is *Ettlia pseudoalveolaris*, a microalga of Ecuadorian origin. While it has been previously studied for its antioxidant, antibacterial, and antimutagenic properties [9], its potential as a biostimulant remains unexplored.

Quinoa (*Chenopodium quinoa* Willd.) is an herbaceous crop native to South America, particularly the Andean regions of Peru, Ecuador, Bolivia, and Chile. For thousands of years, quinoa was cultivated exclusively in these areas, where it served as a staple food for local populations and was revered as the “mother of all seeds” [10]. Since the 1980s, quinoa has gained global recognition due to its exceptional nutritional value, versatility, and genetic diversity. Classified as a pseudocereal, quinoa shares similarities with true cereals in its macronutrient composition, containing carbohydrates (69 g/100 g), protein (16.5 g/100 g), and fats (6.5 g/100 g) [11]. A particularly notable nutritional feature of quinoa is its gluten-free nature, making it an ideal dietary choice for individuals with gluten intolerance or celiac disease [10]. In recognition of its importance, the United Nations General Assembly declared 2013 the “International Year of Quinoa” [12].

Quinoa seeds are rich in bioactive compounds, including phytosterols, phenolic acids, carotenoids, betalains, anthocyanins, and tocopherols. The levels of these compounds can vary depending on factors such as quinoa variety, cultivation site, maturation stage, storage conditions, germination, and exposure to stress [13]. These metabolites serve various functions in plants, such as UV protection, antimicrobial activity, defence mechanisms, and pigmentation of flowers and fruits. Phenolic compounds, characterized by one or more phenolic rings with hydroxyl groups, can exist in free form or bound to cell wall components [14]. The presence of bioactive compounds in quinoa is particularly significant due to their antioxidant properties, which play a crucial role in regulating metabolic processes and reducing inflammation. Carotenoids, a group of pigments produced by plants and algae, are essential for human health as precursors of vitamin A [10]. Polyphenols found in quinoa exhibit antioxidant, anti-inflammatory, and anti-proliferative properties, contributing to the prevention of non-communicable diseases [15]. For instance, phenolic compounds are known to regulate carbohydrate and lipid metabolism by enhancing pancreatic β-cell function and increasing insulin secretion. As a result, polyphenols help lower blood sugar levels, normalize blood lipid profiles, and reduce insulin resistance [16].

The growing global interest in quinoa, driven by its numerous nutritional and nutraceutical benefits, has led to a surge in market demand. Initially, this demand created socio-economic challenges for indigenous populations in the Andean regions [17]. However, it has also spurred the expansion of quinoa cultivation to other parts of the world, including the USA, Canada, Africa, India, China, Denmark, the United Kingdom, France, and Mediterranean regions [13,18]. This widespread adoption has been facilitated by quinoa’s remarkable biodiversity, with approximately 250 different species [11], making it highly adaptable to diverse and extreme environments.

Quinoa exhibits exceptional adaptability, with varieties capable of thriving at altitudes ranging from sea level to over 4000 m. It is a short-cycle crop, reaching maturity in 90–125 days, depending on the sowing season. Quinoa prefers short photoperiods and cooler temperatures for optimal growth and adapts well to silty-sandy soils with a pH range of 4.5 to 8.5 [19]. These traits have made quinoa a focal point of research aimed at developing varieties with even greater resilience to extreme conditions [18,19].

Quinoa is classified as a facultative halophytic crop due to its moderate salt tolerance, withstanding salinity levels of up to 300 mM NaCl [20]. Additionally, it demonstrates tolerance to drought and high temperatures, although the combined stress of drought and heat can significantly reduce crop yields [21].

The aim of this study was to investigate the potential role of microalgae as biostimulants on seven-day-old sprouts of two quinoa varieties with distinct environmental origins (‘Tunkahuan’ and ‘Regalona’). The sprouts were germinated under varying salt concentrations (0, 100, 200, and 300 mM NaCl). Extracts from Chlorella sp. and *Ettlia pseudoalveolaris* were evaluated for their effects on several physiological parameters, including seed germination, seedling growth, production of antioxidant molecules (total phenolics and flavonoids), total antioxidant capacity (measured by DPPH and FRAP assays), induction of reactive oxygen species (ROS) production, and the content of carotenoids, chlorophyll a, and chlorophyll b. These parameters were analyzed to identify the most effective biostimulant and the quinoa variety best suited for stressful environments, as well as to explore the potential production of nutraceutically valuable compounds.

## 2. Results

### 2.1. Effects of Microalgae on Germination and Seedlings Length in Quinoa Exposed to Salt Stress

**Figure 1:**
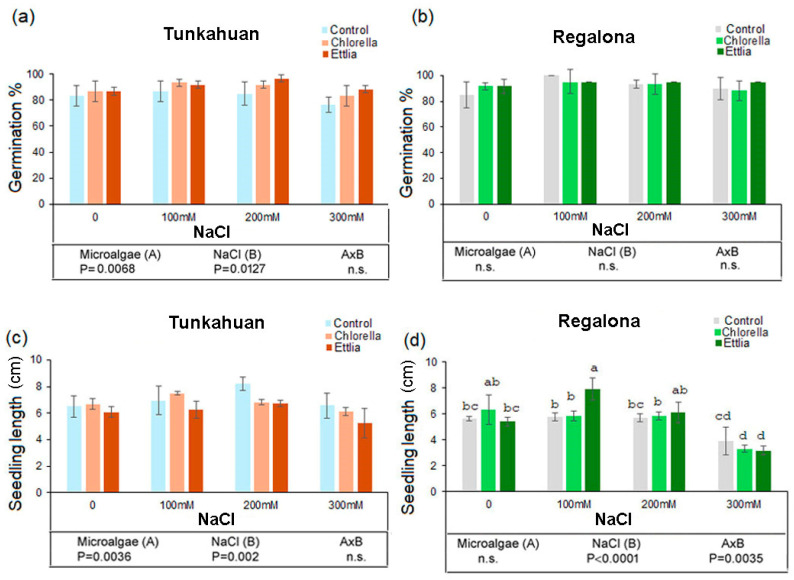
Germination percentage and seedling length in *Chenopodium quinoa* sprouts in ‘Tunkahuan’ (**a**,**c**) and ‘Regalona’ varieties (**b**,**d**) after 7 days from imbibition in presence of *Chlorella* sp. or *Ettlia pseudoalveolaris* (0.05% for both) and NaCl treatments. The values are the mean of three replicates ± SD. Different letters indicate statistically significant differences among the different treatments, according to two-way ANOVA followed by Tukey test (*p* < 0.05). The significance of F test according to the two-way ANOVA is reported in the lower part of the figure.

The percentage of germination was 83.3% in ‘Tunkahuan’ and 85% in ‘Regalona’ in the control condition (Figure 1a,b). Microalgae and salt treatments had a significant effect on var. ‘Tunkahuan’, but not of their interaction (Figure 1). In particular, the treatment with 300 mM NaCl significantly reduced the germination percentage while Ettlia was effective in increasing germination at 200 mM NaCl. No significant effects of microalgae and salt treatments, neither their interaction were observed in var. ‘Regalona’ (Figure 1b).

Microalgae and salt treatments, but not their interaction, had a significant effect on the length of the quinoa seedlings at day 7, in var. ‘Tunkahuan’ (Figure 1c). On the contrary, no significant effect of microalgae was observed in var. ‘Regalona’ (Figure 1d), while salt treatments and salt × microalgae interaction were significant. In particular, 100 mM NaCl with Ettlia extract induced the highest seedling length (7.9 cm) when compared to control (5.7 cm) and treatments with Ettlia or NaCl 100 mM alone (5.4 and 5.8 cm, respectively). Instead, 300 mM NaCl treatment reduced seedling length (3.9 cm in control) and both microalgae had a further negative effect (seedling length about 3.3 cm).

### 2.2. Effects of Microalgae on Total Phenolics and Total Flavonoids Content in Quinoa Sprouts Exposed to Salt Stress

**Figure 2:**
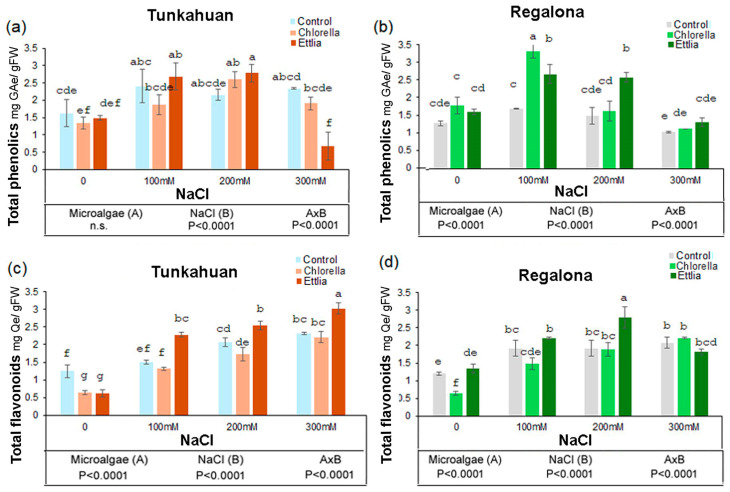
Content of total phenolics and total flavonoids in *Chenopodium quinoa* sprouts in ‘Tunkahuan’ (**a**,**c**) and ‘Regalona’ variety (**b**,**d**) after 7 days from imbibition in presence of *Chlorella* sp. or *Ettlia pseudoalveolaris* (0.05% for both) and NaCl treatments. Values are expressed as mg of gallic acid equivalent/gram of fresh weight (mg GaE/g FW) for phenolics and as mg of quercetin equivalent/gram of fresh weight (mg QE/g FW) for flavonoids The values are the mean of three replicates ± SD. Different letters indicate statistically significant differences among the different treatments, according to two-way ANOVA followed by Tukey test (*p* < 0.05). The significance of F test according to the two-way ANOVA is reported in the lower part of the figure.

In both quinoa varieties NaCl and the interaction between NaCl and microalgae induced significant effects on the content of phenolic compounds, although the microalgae factor was significant only for the ‘Regalona’ variety (Figure 2).

In var. ‘Tunkahuan’, all the concentrations of NaCl induced an increase in the content of these compounds in comparison to the control (Figure 2a).

The effects of microalgae were most noticeable when combined with NaCl. At 100 and 200 mM NaCl an increase in phenolics content with Ettlia and a decrease to 0.68 mg GaE/g FW at the highest NaCl concentration (300 mM NaCl) was observed in comparison to the sample not treated with microalgae and grown without NaCl. For Chlorella an increase, in respect to the control, was observed at 200 mM NaCl. In var. ‘Regalona’, the effect of NaCl was evident at 300 mM with the lowest phenolic content (Figure 2b). The addition of microalgae determined an increase at 100 mM NaCl to 3.34 mg GaE/g FW for Chlorella and to 2.69 mg GaE/g FW for Ettlia in respect to control (1.71 mg GaE/g FW). At 200 mM NaCl, the increase was significant for Ettlia only (2.6 mg GaE/g FW), in respect to control (1.51 mg GaE/g FW). At 300 mM NaCl no effect of microalgae was observed (Figure 2b).

In both quinoa varieties the presence of microalgae and salinity, as well as their interaction had a significant effect on the content of total flavonoids (Figure 2c,d). In ‘Tunkahuan’ variety the control (0 NaCl) sprouts showed the lowest concentration of flavonoids when treated with either Chlorella or Ettlia. With NaCl treatments, a dose-dependent increase was observed in the presence of microalga Ettlia which induced flavonoids to accumulate over the respective control at the same NaCl level; the highest value was observed at 300 mM NaCl (2.95 mg QE/g FW). In quinoa var. ‘Regalona’ the Ettlia-treated sprouts showed a significant increase only at 200 mM NaCl (2.72 mg QE/g FW).

### 2.3. Effects of Microalgae on Antioxidant Activity and ROS Content in Quinoa Sprouts Exposed to Salt Stress

**Figure 3:**
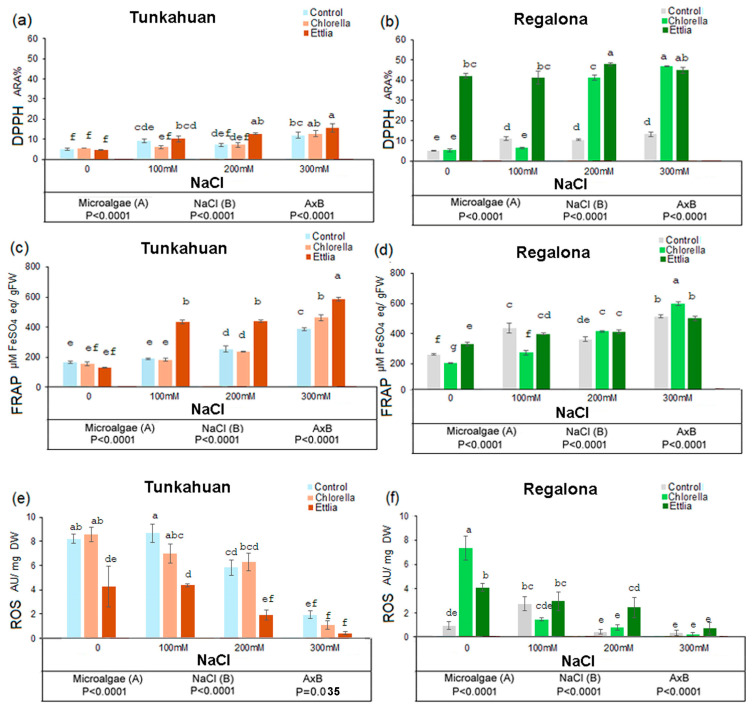
Antioxidant capacity evaluated by DPPH and FRAP assays and determination of ROS level in *Chenopodium quinoa* sprouts in ‘Tunkahuan’ (**a**,**c**,**e**) and ‘Regalona’ variety (**b**,**d**,**f**) after 7 days from imbibition in presence of *Chlorella* sp. or *Ettlia pseudoalveolaris* (0.05% for both) and NaCl treatments. Values are expressed as % of Anti Radical Activity (ARA%) for DPPH assay, as mg FS (ferrous sulphate) equivalents/gram of dry weight (mg FSE/g DW) for FRAP assay, as arbitrary unit/g DW for ROS. The values are the mean of three replicates ± SD. Different letters indicate statistically significant differences among the different treatments, according to two-way ANOVA followed by Tukey test (*p* < 0.05). The significance of F test according to the two-way ANOVA is reported in the lower part of the figure.

The presence of microalgae and salinity, as well as their interaction significantly influenced antioxidant activity, calculated by DPPH assay and by FRAP in both quinoa varieties (Figure 3a,b). In particular, the ‘Tunkahuan’ variety (Figure 3a), showed an increase with raising salinity concentration in the presence of Ettlia (12.72% ARA and 15.73% ARA at 200 mM and 300 mM NaCl, respectively), however this did not happen in the presence of Chlorella. The positive effect of the microalgae was much more evident in ‘Regalona’ variety (Figure 3b), where Ettlia determined an increase in antioxidant activity at all the salt concentrations and in the control, reaching the maximum value at 200 mM and 300 mM NaCl (47.97% and 44.96% ARA, respectively). The addition of Chlorella induced an increase only at 200 mM and at 300 mM NaCl with ARA values of 42.25% and 46.84%, respectively.

The antioxidant activity determined by FRAP assay in the ‘Tunkahuan’ variety significantly enhanced with salinity concentrations of 200 and 300 mM NaCl without biostimulants (Figure 3c). In the presence of Chlorella at 300 mM NaCl, a significant increase was observed (463.66 µM FeSO4 eq/gFW). The treatment with Ettlia, significantly increased the antioxidant activity at all the salt concentrations, reaching the highest value of 586.16 µM FeSO4 eq/gFW at 300 mM NaCl. Similarly, for the ‘Regalona’ variety (Figure 3d), the highest antiradical activity was recorded for all the samples at a salinity concentration of 300 mM, the highest value occurring in the presence of Chlorella (565.33 µM FeSO4 eq/gFW) (Figure 3d).

The concentration of ROS in both quinoa varieties was significantly impacted by salt and microalgae, as well as their interaction (Figure 3e,f). The addition of Ettlia, but not Chlorella, was effective in lowering ROS levels in the control samples, and in 100 and 200 mM NaCl in the ‘Tunkahuan’ variety (Figure 3e). The 300 mM NaCl significantly reduced ROS level in all the ‘Tunkahuan’ samples. In the ‘Regalona’ variety (Figure 3f) ROS were extremely low in the control sample (0.88 AU/mg DW) but much higher in the presence of Chlorella (7.22 AU/mg DW) and of Ettlia (4.00 AU/mg DW). The presence of salt significantly reduced ROS content in the presence of microalgae, with values of 1.38, 0.74 and 0.16 AU/mg DW in Chlorella samples and 2.9, 2.4, 0.6 AU/mg DW in Ettlia at 100, 200 and 300 mM NaCl, respectively.

### 2.4. Effects of Microalgae on Pigments Content in Quinoa Sprouts Exposed to Salt Stress

HPLC analysis revealed that chlorophyll *a* and *b* increased with rising salinity concentrations both with and without biostimulants. However, the highest content of these two pigments was always found in the control samples, not added with the biostimulants. The highest content of chlorophyll *a* and *b* was recorded at 200 mM NaCl in the absence of biostimulants for the ‘Tunkahuan’ variety (930 µg/g DW for chlorophyll *a* and 550 µg/g DW for chlorophyll *b*) (Table 1), and at 100 mM (1693.59 µg/g DW for chlorophyll *a*, 910.56 µg/g DW for chlorophyll *b*) and at 200 mM NaCl (1685.9 µg/g DW for chlorophyll *a*, 827.92 µg/g DW for chlorophyll *b*) in the absence of biostimulants for the ‘Regalona’ variety (Table 2).

Additionally, β-carotene and lutein were also quantified. In both quinoa varieties, these pigments were generally more prevalent in the absence of biostimulants, with the highest values recorded at 200 mM as already observed for the two chlorophylls. However, the content of lutein and β-carotene in ‘Regalona’ at 200 mM NaCl was not influenced by the addition of the algal biostimulants. Specifically, in the ‘Tunkahuan’ variety (Table 1), the content of β-carotene at 200 mM without biostimulants was 78.81 µg/g DW, while the lutein content was 470.43 µg/g DW. In the ‘Regalona’ variety (Table 2), the content of β-carotene at 200 mM in without biostimulants was 122 µg/g DW, while the lutein content was 792.03 µg/g DW.

### 2.5. Canonical Discriminant Analysis (CDA) and Pearson’s Correlation

**Figure 4:**
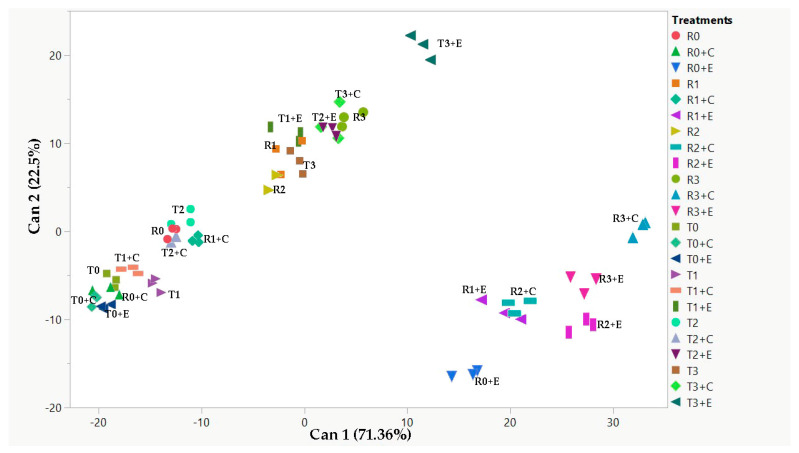
2D scatterplot of canonical discriminant analysis. Can 1 and 2 refer to canonical function 1 and 2, which consider all variables in order to maximize the separation between the groups. T = Tunkahuan, R = Regalona, C = Chlorella, E = Ettlia, 0, 1, 2, 3 = 0, 100, 200, 300 mM NaCl.

Canonical discriminant analysis (CDA) was performed, as shown in Figure 4. Additionally, four MANOVA tests (Wilk’s Lambda, Roy’s Largest Root Test, Hotelling-Lawley Trace, and Pillai-Bartlett Trace) were conducted to assess whether the groups differed based on the given variables. In this CDA, all MANOVA tests yielded highly significant results (*p* < 0.0001), indicating the robustness of the group separation. Furthermore, Pearson’s correlation coefficients were calculated between shoot length, germination percentage, total phenolics and total flavonoids content, total antioxidant activity assessed by DPPH and FRAP assays, and ROS content, to identify which individual variables were the main contributors to the group separation. This CDA was performed considering both quinoa varieties in the presence or absence of salt stress (0 mM, 100 mM, 200 mM, and 300 mM NaCl), in the presence or absence of the two biostimulants (*Chlorella vulgaris* and *Ettlia pseudoalveolaris*).

Canonical 1, which can be related to the saline treatment, explained most of the separation (71.36%), while canonical 2, which can be related to the presence of biostimulants, accounted for 22.5% of the separation of our samples.

CDA analysis allowed the separation of the two varieties mainly in function of salt and biostimulant treatment (Figure 4).

Pearson’s correlation (Table 3) revealed that shoot length and the presence of ROS are negatively correlated with the presence of salt, unlike total antioxidant activity, which is positively correlated by DPPH and FRAP assays. In the case of canonical function 2, a positive correlation between the presence of biostimulants and total antioxidant activity measured by the FRAP assay was observed.

## 3. Discussion

In the age of climate change marked by increasing temperatures, extreme weather events, drought and consequent soil salinity, it is crucial to analyze the response of plants to salt stress [2] and to suggest novel strategies like using biostimulants to increase plant resistance to adverse conditions [7,22]. The halophytic quinoa emerges among crop plants as one of the most promising species to be introduced in marginal lands because of its genetic resilience and adaptability. Quinoa represents a resource to ensure food security and environmental sustainability in the Mediterranean regions that are increasingly impacted by climate change [23].

The results of this study, which examined two quinoa varieties exposed to salt stress, indicated that quinoa germination was unaffected by microalgae co-treatments as biostimulant. Instead, in ‘Regalona’ seedling length was significantly influenced by Ettlia microalgae with an increase at 100 mM and an impairment at 300 mM NaCl.

Our results indicated that ‘Regalona’ was more sensitive to Ettlia biostimulant in line with recent research showing beneficial effects of microalgae as growth additives on plant systems [24], including quinoa [25].

Phenolic compounds, one of the most abundant groups of secondary metabolites in plants, play a key role in numerous metabolic and physiological processes. Indeed, abiotic stress often triggers their synthesis and accumulation [26].

The overall rise in phenolic compounds found in the ‘Tunkahuan’ variety in response to salt treatment is consistent with earlier research [20]. No statistically significant changes were observed in relation to salt treatment following the addition of microalgae, with the exception of a decrease in phenolic compounds in the presence of Ettlia at the highest salt concentration (300 mM NaCl). Nevertheless, in the ‘Regalona’ variety, a significant rise in phenolic compounds was observed in presence of Chlorella at NaCl 100 mM, and in the presence of Ettlia at the concentrations of 100 and 200 mM NaCl. This suggests that the effect of the microalgae was variety- and salt-dependent. The reduced phenolic compounds in ‘Tunkahuan’ variety at 300 mM NaCl, indicated that the Ettlia biostimulant alleviated salt-induced stress in this quinoa variety, while no effect was observed in ‘Regalona’ variety.

A plant’s adaptive response to abiotic stress was demonstrated by the dose-dependent rise in total flavonoid content induced by salt stress in both quinoa varieties. The ‘Tunkahuan’ variety exhibited a steady rising trend in flavonoid levels at all salt concentrations, even in the presence of Ettlia, whereas the ‘Regalona’ variety only showed a noticeable increase at 200 mM NaCl. According to these results, ‘Tunkahuan’ may have a stronger ability to accumulate flavonoids under varying salt conditions, which could explain its higher resilience against oxidative stress.

The analysis of the total antiradical capacity (ARA%) by the DPPH assay revealed that the two quinoa varieties behaved differently. ‘Tunkahuan’ showed a moderate increase in antioxidant activity at 100 and 200 mM NaCl when treated with Ettlia, with the antioxidant activity being approximately 10%. In contrast, ‘Regalona’ exhibited a more pronounced response, with a significant increase with Chlorella at doses of 200 and 300 mM NaCl and with Ettlia in all treatments considered, reaching levels of about 60% ARA. These results suggest the potential of microalgae to enhance oxidative stress tolerance, indicating that ‘Regalona’’s antioxidant system is highly responsive to biostimulant treatments.

Regarding the reducing power, which was evaluated using the FRAP assay, both quinoa varieties showed a dose-dependent increase in this activity when treated with NaCl. The antioxidant activity was increased by co-treatment with microalgal extracts. In the ‘Tunkahuan’ variety, Chlorella only significantly improved the reducing power at 300 mM NaCl, while Ettlia produced a significant increase across all salt treatments. In the ‘Regalona’ variety, the most significant effects were observed with co-treatment of Chlorella and NaCl 200 and 300 mM, as well as with Ettlia without salt and at 200 mM NaCl. These findings highlight the different responses of the two quinoa varieties to microalgae, with ‘Tunkahuan’ responding to Ettlia more reliably.

The results on ROS content further highlighted the contrasting behaviors of the two quinoa varieties. Under control conditions, the ‘Tunkahuan’ variety showed higher basal ROS levels than ‘Regalona’. Interestingly, Chlorella did not significantly alter the ROS levels in either variety, except in the control of ‘Regalona’, where it appeared to induce a non-negligible stress. In comparison to the controls, the presence of Ettlia induced a reduction in ROS content in the ‘Tunkahuan’ variety, and an increase in the ‘Regalona’. These observations suggested that Ettlia’s impact on ROS content is variety-specific, possibly reflecting differences in metabolic and stress-response pathways in the two quinoa genotypes.

The results highlight the complex and variety-dependent impact of salt stress and microalgae treatments on quinoa, emphasizing their role in modulating antioxidant responses and managing oxidative stress.

This supports earlier studies on the protective effects of biostimulants and further reinforces the role of microalgae as valuable tools in sustainable agriculture [27]. Previous studies on chlorella demonstrated its capacity to improve wheat growth when applied as aqueous extract, filtered culture, crude culture and sonified culture in soil treatments. Interestingly, both the dose and the type of *C. vulgaris* treatment could positively influence the growth parameters of the plants [28]. Chlorella produces a wide range of molecules that are responsible for these effects including organic and inorganic compounds like polysaccharides, lipids and fats, proteins, vitamins, macro and micronutrients, in addition to phytohormones such as auxin, abscissic acid, cytokinins and gibberellins which can enhance plant growth even under stress conditions [29].

The species and strain of microalgae may potentially interfere with the biostimulant effects on different plants, Chlorella being one of the most effective microalga to counteract adverse environmental stressors, such as drought, salt, cold and heat [30,31].

In our experiment, Ettlia in particular, had a remarkable ability to reduce oxidative stress and enhance antioxidant responses, mainly in ‘Tunkahuan’ variety. Recently, this microalga has been characterized by demonstrating the presence of bioactive compounds with nutraceutical properties (antioxidant, antibacterial and antimutagenic action, as well as lipids, polyunsaturated fatty acids) [9]. The presence of antioxidant molecules could explain the protective and biostimulant action of Ettlia in stressed plants.

Previous study demonstrated that the enhanced activity of the enzyme PAL (Phenylalanine ammonia-lyase) caused the prompt activation of secondary metabolism, which plays a role in plant stress tolerance. This enzyme is implicated in the phenylpropanoid pathway for the synthesis of phenolics and flavonoids, which are the principal molecules involved in plant defence responses for their antioxidant properties contrasting ROS caused by salt stress [24,32]. Recent research indicated that polysaccharide extracts obtained from different microalgal strains also increased the activity of antioxidant enzymes such as catalase, peroxidase, and superoxide dismutase in plants under salt stress [4]. All mechanisms may be devoted to reduce the ROS level in plant cell and to counteract the oxidative damage and lipid peroxidation, improving membranes and proteins integrity [5].

The analysis of chlorophyll and carotenoid content provided valuable insights into the physiological responses of quinoa varieties ‘Tunkahuan’ and ‘Regalona’ to the presence of biostimulants at different salinity levels. The results demonstrated an increase in chlorophyll *a* content with increasing saline concentration in both varieties. The highest levels were consistently observed in control samples devoid of biostimulants, indicating that their addition negatively impacted chlorophyll *a* accumulation under saline conditions. An exception was observed in the ‘Regalona’ variety at 200 mM NaCl, where Chlorella had no negative impact on chlorophyll *a* content. A similar behavior in both quinoa varieties was observed for chlorophyll *b*, which rose with increasing salt concentration and was reduced by the presence of the biostimulants except in ‘Regalona’ at 200 mM. Lastly, it was shown that β-carotene and lutein were more abundant in quinoa sprouts in the absence of biostimulants in both varieties, except in ‘Regalona’ at the highest salt level, and that their amount increased as the concentration of NaCl increased. This pattern is consistent with the results about chlorophylls. Controversial results were reported in the literature about how environmental stressors affect chlorophyll content, with some reports indicating a decrease [33] or an increase [34] in photosynthetic pigments. Specifically, the sensitivity of the plant ecotype seems to influence the responses of chlorophyll and carotenoid to salinity stress [35].

Our findings suggest that ‘Tunkahuan’ and ‘Regalona’ both exhibit a moderate tolerance to salinity, as evidenced by their ability to retain or even increase pigment levels under stressful conditions. Although biostimulants like Chlorella and Ettlia might provide other advantages, their impact on photosynthetic pigments in saline conditions appears limited or even detrimental. There might be several physiological and biochemical factors for this adverse effect. One plausible explanation is that sprouts prioritize some metabolic processes over others due to their rapid growth phase, and the presence of biostimulants may shift metabolic resources away toward stress responses or antioxidant production, rather than to pigment synthesis. Because biostimulants may induce mild oxidative stress in sprouts due to the presence of reactive metabolites or hormonal imbalances, this metabolic shift could explain the reduction in pigment content. Indeed, in the two quinoa varieties studied, changes in the amount of bioactive compounds (phenolics and flavonoids) and in antioxidant activity resulted from microalgae addition. Biostimulants can also alter the availability of key nutrients (e.g., nitrogen, magnesium, or iron) [36] essential for the biosynthesis of chlorophyll. In sprouts, where nutrient availability and utilization are crucial for development, even slight imbalances may result in reduced pigment production.

Previous works have highlighted that Chlorella microalgae treatment can improve plant growth parameters and boost drought tolerance under water deficient circumstances by enhancing secondary metabolites, nutritional contents, and antioxidant enzyme activities [37,38]. Quinoa has demonstrated excellent adaptability to a wide range of habitats and growing conditions, both for the intrinsic variability of its germplasm and the varietal selection carried out by producers since ancient times [18,39].

In this work, the impact of two algal biostimulants on quinoa germination and bioactive compounds in sprouts under salt stress conditions was examined; however, future research should examine other environmental stresses and their combinations, such as drought and temperature, which are always connected to climate change. In fact, these environmental elements can combine to provide adverse conditions for plant development [40], which is also influenced by increased virulence and plant diseases transmission [41].

Given the accelerating climate change which may disadvantage the cultivation of other crops, quinoa cultivation, as a species resistant to a variety of abiotic stresses, may play a fundamental role in human and animal nutrition. The use of biostimulants could further increase quinoa performance under environmental stressors such as drought, high temperatures and salt stress.

## 4. Materials and Methods

### 4.1. Plant Material and Treatments

In the present research, seeds of two quinoa varieties, *Chenopodium quinoa* Willd. ‘Tunkahuan’, cultivated in Ecuador and *Chenopodium quinoa* Willd. ‘Regalona’, cultivated in Italy, were selected based on their different environmental origin in order to compare their response to treatments with biostimulants and salt stress. Quinoa Seeds of the variety ‘Tunkahuan’ were provided by the Ecuador’s National Institute of Agricultural Research (INIAP) and the variety ‘Regalona’ was kindly provided by the company Tuttoquinoa, Manciano (GR) Italy. Seeds of the two varieties were germinated in 9 mm Petri dishes on filter paper wetted with 12 solutions as a result of interactions between the salt treatments with NaCl (0, 100, 200, 300 mM corresponding to EC 0, 10, 20, 30 dS·m^−1^) and microalgae extracts (control, 0.05% *Ettlia pseudoalveolaris*, 0.05% *Chlorella vulgaris* extracted for 4 h at room temperature). In each Petri dish, 25 seeds of quinoa ‘Tunkahuan’ and quinoa ‘Regalona’ were germinated, making 4 replicates for a total of 100 seeds per each experimental thesis. For germination tests, a 3-day dark period was followed by a 4-day light exposure in germinating chambers set at 24 °C. The seedlings were analyzed at 7 days, which corresponds approximately to the 08-09 BBCH stage (Hypocotyl with cotyledons growing towards soil surface-emergence of cotyledons through soil) [42] for germination percentage and length.

### 4.2. Preparation of Quinoa Extracts

One gram of seedlings of the two quinoa varieties was placed in 10 mL 80% EtOH. The samples were then homogenized using an Ultra Turrax homogenizer (Kinematica Polytron PT MR 2100, Lucerne, Switzerland) and extracted on a shaker overnight. After, the tubes were centrifuged at 3500× *g* for 30 min at 4 °C with Jouan CR31 centrifuge, Newport Pagnell, UK. The supernatant was either used for the various spectrophotometric assays or stored at −20 °C. Three replicates of each of the following determinations were carried out using a UV/visible spectrophotometer (Perkin Elmer, Victor TM X3 apparatus, Waltham, MA, USA).

### 4.3. Determination of Total Phenolics Content

The total phenolics content was determined by a colorimetric assay based on the Singleton et al. method [43]. Fifty µL of plant extract and 1 mL of Folin-Ciocalteau (Merck, Sigma-Aldrich, GmbH, Sternheim, Germany) (diluted 5 times with distilled water) were added to a cuvette and incubated in the dark at room temperature (RT) for 6 min. Subsequently, 660 µL of 20% Na_2_CO_3_ were added, and the mixture was incubated in the dark for one hour at RT. The absorbance was measured at 760 nm against a blank of 80% EtOH. The results are expressed in mg of gallic acid equivalents per gram of fresh weight (mg GaE/g FW).

### 4.4. Determination of Total Flavonoid Content

Total flavonoid content was evaluated by the modified method of Heimler et al. [44]. Briefly, to 150 µL of plant extract, 600 µL of distilled water, and 45 µL of 5% NaNO_2_ were added, and the mixture was incubated in the dark at RT for 5 min. After that, 45 µL of 10% AlCl_3_ was added and the mixture incubated for 6 min. Following the addition of 300 µL of 1 M NaOH and of 360 µL of distilled water, the samples were incubated in the dark for 30 min. Lastly, a spectrophotometric analysis was performed at 430 nm against 80% EtOH as blank. The results are expressed in mg of quercetin equivalents per gram of fresh weight (mg QE/g FW).

### 4.5. DPPH Assay

DPPH (2,2-Diphenyl-1-picrylhydrazyl) (Merck, Sigma-Aldrich, GmbH, Sternheim, Germany) assay was based on the modified method by Boudjou et al. [45]. An 80 µM DPPH solution in methanol 80% was stirred for one hour in the dark. Fifty µL of plant extract and 1470 µL of DPPH were incubated in the dark for one hour. Absorbance was measured at 517 nm against 80% EtOH as blank. Antiradical activity (ARA) is expressed as the percentage inhibition of DPPH and calculated using the formula:

ARA = 100 × [1 − (Abs of sample/Abs of control)]

### 4.6. FRAP Assay

The assay, based on the modified method by Benzie and Strain [46], determined the antioxidant capacity by measuring the reduction of ferric (Fe^3+^) to ferrous (Fe^2+^) ions at low pH levels. Fifty-one µL of plant extract were added to 1.5 mL of FRAP buffer containing TPTZ (10 mM in 40 mM HCl), FeCl_3_·6H_2_O (20 mM), and acetate buffer (300 mM, pH 3.6). The mixture was incubated in the dark at RT for one hour, subsequently measured by spectrophotometric analysis at 593 nm. The FRAP activity of the plant samples was calibrated against a standard curve of ferrous sulfate (Fe_2_SO_4_·7H_2_O). Results were expressed as µM FeSO_4_ equivalents/g FW.

### 4.7. Quantification of ROS (Reactive Oxygen Species)

The quantification of ROS was performed placing 50 mg of sprouts in 100 mM 2′,7′-dichlorofluorescein diacetate (DCFDA) in 40 mM TRIS HCl buffer, shaken in the dark at 37 °C for 60 min. After, the sprouts were washed with distilled water, surface dried, weighed, put overnight at 60 °C and weighed again to obtain the dry weight. The quantification of ROS was performed by measuring the fluorescence emitted by the oxidized DCFDA at 488 nm and 525 nm. The results were expressed in arbitrary units (AU) per milligram of dry weight [47].

### 4.8. HPLC (High Performance Liquid Chromatography)

The lyophilized samples were prepared by weighing 0.05 g of powder and extracting them with 1 mL of 80% acetone for 30 min at 4 °C in the dark. After centrifugation and recovering of the supernatant, the pellet was extracted with 1 mL 100% acetone as described above. The two supernatants were merged and filtered using 0.2-μm filters (Sartorius Stedim Biotech, Goettingen, Germany). The filtered supernatant was analyzed using a Vanquish HPLC system equipped with the Vanquish Diode Array Detector CG (Thermo Fisher Scientific, Waltham, MA, USA). A Prodigy C18 column (SA, 5-μm particle size, 250 × 4.6 mm; Phenomenex, Castel Maggiore, Italy) was used to separate the compounds using a mobile phase of acetonitrile/methanol (75/25 *v*/*v*) and methanol/ethyl acetate (68/32 *v*/*v*) [48]. The flow rate was 1 mL/min, with an injection volume of 20 µL. Data were analyzed using Chromeleon 7.3.1 software (Thermo Fisher Scientific, Waltham, MA USA).

### 4.9. Statistical Analysis

The experimental data were analyzed using two-way ANOVA, and the Tukey test (*p* ≤ 0.05) was used to compare the effects of two factors: treatments with NaCl, treatments with microalgae and their interaction, identifying significant differences. Statistical analysis was performed using JMP software (JMP, Version 17—SAS Institute Inc., Cary, NC, USA). Results were reported as the mean of the three replicates with standard deviation (±SD). Different letters were used to label treatments that differed significantly.

A Canonical Discriminant Analysis (CDA) was conducted across all experimental groups, incorporating all parameters examined in this study, including shoot length, germination percentage, total phenolic and flavonoid concentrations, total antioxidant activity (DPPH and FRAP methods), and ROS content. Additionally, a Pearson’s correlation analysis was performed between the variables and the canonical scores from the first and second canonical functions.

## 5. Conclusions

In conclusion, our analysis clearly indicates that Ettlia microalgae significantly impacted the parameters of interest more than Chlorella. Ettlia treatments determined a more consistent increase in the total content of polyphenols and flavonoids in the two quinoa varieties ‘Tunkahuan’ and ‘Regalona’ in NaCl co-treatment, while Chlorella had a less noticeable effect, particularly in the ‘Tunkahuan’ variety. Furthermore, Ettlia treatments increased total antioxidant activity and reducing power, suggesting that Ettlia extract has a greater capacity to promote neutralization of free radicals, as seen by the ROS reduction in the ‘Tunkahuan’ variety.

These results are very encouraging for further studies and for the application of Ettlia microalgae as biostimulant in quinoa cultivation and in presence of salt stress. Moreover, the significant increase in bioactive compounds in the treated plants led to increased resistance and nutraceutical benefits. As an enriched food that can be eaten as sprout or as dietary supplement, quinoa could then become a significant resource in the diet of the future.

## Figures and Tables

**Table 1:** Concentration of lutein, β-carotene, chlorophyll *a* and *b* by HPLC analysis of freeze-dried sprouts of *Chenopodium quinoa* variety ‘Tunkahuan’ in the presence or absence of microalgae of *Chlorella* sp. or *Ettlia pseudoalveolaris* (0.05% for both) and NaCl treatments. The values expressed as µg/g DW are the mean of three replicates ± SD. The presence of different letters indicates significantly different values according to the Tukey test (*p* ≤ 0.05). The significance of F test according to the two-way ANOVA is reported in the lower part of the table.

NaCl		Lutein	β-Carotene	Chlorophyll *a*	Chlorophyll *b*
0 mM	Control	121.93 ± 4.17 d	16.42 ± 0.97 f	196.64 ± 22.33 e	143.77 ± 0.93 e
	Chlorella	64.19 ± 8.5 e	0 g	94.01 ± 13.9 f	66.99 ± 9.75 f
	Ettlia	71.38 ± 3.36 e	0 g	119.19 ± 0.93 ef	75.5 ± 0.79 f
100 mM	Control	320.39 ± 1.6 b	49.61 ± 2 c	544.53 ± 15.65 c	381.8 ± 16.21 b
	Chlorella	151.43 ± 4.29 cd	23.09 ± 0.76 e	318.7 ± 2.94 d	187.2 ± 3.15 de
	Ettlia	189.75 ± 10.15 c	28.93 ± 1.04 d	338.33 ± 13.68 d	201.32 ± 11.65 d
200 mM	Control	470.43 ± 5.46 a	78.81 ± 0.82 a	930.51 ± 28.2 a	552.08 ± 10.09 a
	Chlorella	341.03 ± 16.84 b	58.39 ± 2.07 b	690.88 ± 33.19 b	387.21 ± 20.43 b
	Ettlia	313.53 ± 17.01 b	50.84 ± 2.09 c	568.94 ± 33.84 c	310.97 ± 18.52 c
ANOVA					
Microalgae (A)		*p* < 0.0001	*p* < 0.0001	*p* < 0.0001	*p* < 0.0001
NaCl (B)		*p* < 0.0001	*p* < 0.0001	*p* < 0.0001	*p* < 0.0001
A × B		*p* = 0.0002	*p* = 0.0002	*p* < 0.0001	*p* < 0.0001

**Table 2:** Concentration of lutein, β-carotene, chlorophyll *a* and *b* by HPLC analysis of freeze-dried sprouts of *Chenopodium quinoa* variety ‘Regalona’ in the presence or absence of microalgae of *Chlorella* sp. or *Ettlia pseudoalveolaris* (0.05% for both) and NaCl treatments. The values expressed as μg/g DW are the mean of three replicates ± SD. The presence of different letters indicates significantly different values according to the Tukey test (*p* ≤ 0.05). The significance of F test according to the two-way ANOVA is reported in the lower part of the table.

NaCl		Lutein	β-Carotene	Chlorophyll *a*	Chlorophyll *b*
0 mM	Control	389.44 ± 0.83 d	63.85 ± 1.96 c	889.76 ± 1.35 d	479.57 ± 6.57 e
	Chlorella	265.31 ± 11.52 e	36.57 ± 0.3 d	593.16 ± 34.5 e	363.73 ± 16.96 cd
	Ettlia	253.49 ± 31.98 e	25.58 ± 1.64 d	494.47 ± 115.43 e	282.06 ± 58.55 f
100 mM	Control	687.81 ± 35.7 b	118.3 ± 2.1 a	1693.59 ± 78.4 a	910.56 ± 43.35 a
	Chlorella	514.22 ± 28.98 c	78.63 ± 5.18 c	1186.23 ± 8.81 c	675.39 ± 8.54 cd
	Ettlia	502.39 ± 21.52 c	86.41 ± 1.61 bc	1179.25 ± 58.18 c	607.65 ± 28.62 d
200 mM	Control	792.03 ± 16.8 a	122 ± 1.61 a	1685.9 ± 31.38 a	827.92 ± 17.92 ab
	Chlorella	752.23 ± 15.46 ab	121.03 ± 0.49 a	1598.41 ± 5.5 ab	786.75 ± 2.96 bc
	Ettlia	716.89 ± 30.5 ab	105.77 ± 2.87 ab	1439.16 ± 63.05 b	725.74 ± 34.02 bc
ANOVA					
Microalgae (A)		*p* < 0.0001	*p* < 0.0001	*p* < 0.0001	*p* < 0.0001
NaCl (B)		*p* < 0.0001	*p* < 0.0001	*p* < 0.0001	*p* < 0.0001
A × B		*p* = 0.0247	*p* = 0.0106	*p* = 0.0078	*p* = 0.0063

**Table 3:** Pearson’s correlation coefficients (r) between shoot length, germination percentage, total phenolics, total flavonoids, total antioxidant activity (DPPH and FRAP), ROS content, and the canonical scores from the canonical discriminant analysis (CDA) reported in Figure 4. In the CDA, the canonical scores from canonical function 1 and 2 were considered, explaining 71.36% and 22.5%, respectively.

Pearson’s Coefficient		
	Can 1	Can 2
Seedling length	−0.53 *	−0.21
Germination %	0.15	−0.10
Total phenolics	−0.14	−0.13
Total flavonoids	0.45	0.46
DPPH	0.75 *	−0.35
FRAP	0.67 *	0.56 *
ROS	−0.61 *	−0.48

* 0.5 > |r| > 0.8: strong correlation.

## Data Availability

The original contributions presented in the study are included in the article; further inquiries can be directed to the corresponding author.
